# Proper oral hygiene protocols decreased inflammation of gingivitis in a patient during chemotherapy with bevacizumab: a case report

**DOI:** 10.1002/ccr3.1034

**Published:** 2017-07-08

**Authors:** Kazuyo Mori, Miho Horinouchi, Ayumi Domitsu, Takako Shimotahira, Sakiko Soutome, Taihei Yamaguchi, Takahiko Oho

**Affiliations:** ^1^ Division of Clinical Engineering Kagoshima University Hospital Kagoshima Japan; ^2^ Perioperative Oral Management Center Nagasaki University Hospital Nagasaki Japan; ^3^ Department of Preventive Dentistry Research Field in Dentistry, Medical and Dental Sciences Area Kagoshima University Kagoshima Japan

**Keywords:** Adverse effects, bevacizumab, chemotherapy, gingivitis, ovarian cancer

## Abstract

The case is a woman who had a diagnosis of ovarian cancer and endometrial cancer. After surgical therapy, platinum‐based adjuvant treatment was performed, followed by additional bevacizumab administration. Because considerable gingivitis appeared, a proper approach for oral hygiene was performed. As a result, the symptom was reduced considerably.

## Introduction

Epithelial ovarian cancer is one of the fatal gynecologic malignancies. Patients with stage I or II are generally asymptomatic because the organ is present inside pelvis. The stage progressed to III or IV, and a malignant lesion has spread to the upper abdomen already in most patients when the disease is detected. The first‐line treatment for these patients is cytoreductive surgery, followed by chemotherapy, with a combination of platinum and paclitaxel 1. When patients experienced recurrence over 6 months after the completion of the initial treatments, their disease is estimated as platinum‐resistant or platinum‐refractory. Although single‐drug regimens, including pegylated liposomal doxorubicin, gemcitabine, or topotecan, are indicated for these cases, standard treatment has not been established yet 2.

The amount of vascular endothelial growth factor (VEGF) was reported to be associated with ascites formation and poor prognosis in ovarian cancer 3. Bevacizumab is a molecular‐targeted agent, which is a recombinant humanized monoclonal IgG antibody. It works against VEGF and inhibits angiogenesis by neutralization of biologic properties of VEGF 4. The addition of the bevacizumab to conventional administration of carboplatin and paclitaxel chemotherapy improved progression‐free survival significantly for patients with advanced or poor‐prognosis early‐stage ovarian cancer 5, 6. In 2014, a combination of bevacizumab and conventional administration of carboplatin and gemcitabine in chemotherapy provided improvement in progression‐free survival for patients with recurrent ovarian cancer in randomized trials 7. There are some reports with advance results for other kinds of malignant lesions also by bevacizumab. Survival rate was much greater in patients prescribed with standard chemotherapy regimens added by bevacizumab than only conventional regimen for brain 8, metastatic breast 9, colorectal 10, and nonsmall cell lung cancer 11.

On the other hand, hypertension, proteinuria, and thrombosis appeared frequently, as incidence of adverse effects by the administration of bevacizumab. Gastrointestinal disorders appear frequently, and the incidence of gastrointestinal perforation was higher than reported in bevacizumab trials of other tumor types (5/44) in a phase II study of platinum‐resistant ovarian cancer or peritoneal serous cancer. The highest possible risk factor for gastrointestinal perforation was suggested an application of three prior chemotherapy regimens 12. In other study for recurrent ovarian cancer (n = 15) prescribed with bevacizumab plus cyclophosphamide, neither gastrointestinal perforation nor fistulas was detected. Two patients had grade I epistaxis and gingival bleeding 13. To date, the effect of antiangiogenic agents like bevacizumab for oral bleeding has not been investigated well.

In the present study, a case is described in which gingiva was indicated symptoms of inflammation including swelling and bleeding, induced by administration of bevacizumab. Moreover, poor oral hygiene made the symptoms worse. Introduction of a proper dental hygiene program during the chemotherapy brought improvement to the oral symptom significantly.

## Case Report

A 44‐year‐old Japanese woman visited the dental clinic office of the Oral Care Center in Kagoshima University Hospital according to a suggestion by her gynecologist. She complained about pain and bleeding from gingiva by routine toothbrushing. She was diagnosed with ovarian cancer (stage IIIc) and received surgical therapy to remove all of the ovarian and surrounding lymph nodes, including the most malignant lesion. This was followed by chemotherapy to destroy the remaining cancer cells on the 15th day after the surgery. Anticancer drugs selected first were paclitaxel and carboplatin as a combination regimen. This administration was repeated six times approximately every 3 weeks. In addition, bevacizumab was also prescribed in the same day from the second to sixth course, and only bevacizumab was injected an additional 16 times after that. Finally, the total times of prescription during this chemotherapy were 21.

On the third day after the first injection, general adverse effects, which were urticarial, flare, and swelling in part, emerged, and she had strange feeling around the gingiva as an oral adverse effect as well. Although a combination regimen of docetaxel and carboplatin was administrated only in the third course, general adverse effects emerged similarly. Fortunately, these general effects have decreased gradually, but the oral problem was remaining and exacerbated gradually in contrast. During the whole period of chemotherapy, the patient did not experience hypertension, proteinuria, thrombosis, epistaxis, or gastrointestinal perforation as a major adverse effect of bevacizumab.

**Figure 1:**
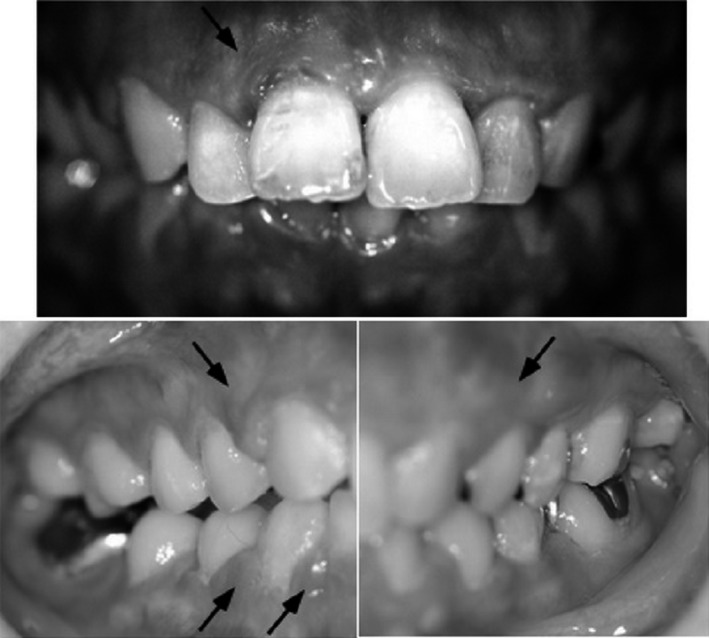
Clinical appearance at the first visit of dental office in the 11th course of chemotherapy. Swelling of gingiva as a symptom of inflammation was scored as grade 2–3. Other oral mucositis and xerostomia were not detected. Arrows denote edematous gingiva associated with serious hemorrhage.

On the day of the 11th course of chemotherapy, which was the sixth course of only bevacizumab, the patient visited a dental office for the first time and complained about the oral disorder (Fig. 1). Oral examination revealed followings: total number of tooth was 28; DMFT was 16 (16 filled teeth); gingival swelling was scored as grade 2–3 according to Gingival Index (GI); and edematous gingiva associated with serous hemorrhage was detected in many parts. Extensive oral mucositis and xerostomia were not detected. Plaque Control Record (PCR) value as oral contamination was 82.1%. Periodontal probing depths were generally 4 mm or more. Most sites of deep probing depth were bleeding in response to periodontal probing. There were no moving teeth or occlusal trauma. The area of attached gingiva was not a problem in any teeth. The patient was unable to brush her teeth due to fear of pain and bleeding from gingiva for a long time until the first visit to the dental office.

**Figure 2:**
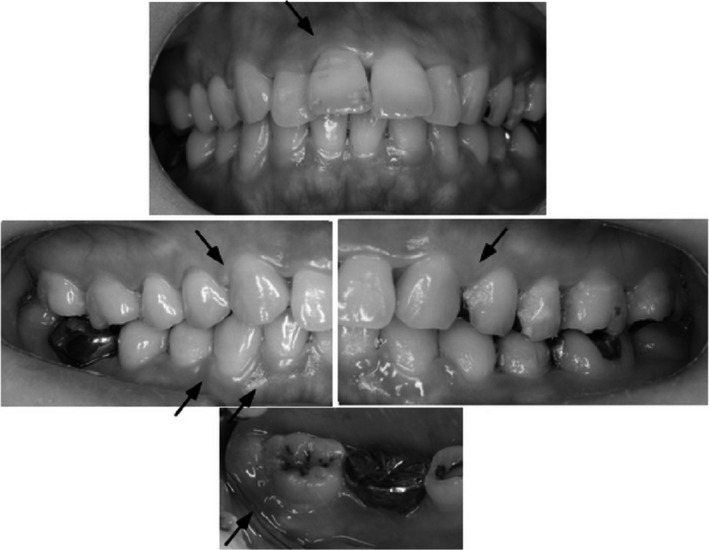
Clinical appearance after 4 weeks from the end of chemotherapy. Arrow denotes edematous gingiva associated with serous hemorrhage, which localized around lower left second molar.

The symptoms, which were bleeding and swelling of gingiva, emerged approximately 1 week later from every injection of bevacizumab and lasted for next 10 days. We informed her that the gingival symptoms were derived from insufficient cleaning and bad oral contamination as well as the chemotherapy itself, primarily. Appropriate oral hygiene techniques were indicated, and professional mechanical tooth cleaning (PMTC) was carried out focusing on only supragingival areas to avoid pain and bleeding. The patient brushed her teeth using a very soft‐bristle toothbrush and an end‐tuft brush for implanted teeth three times or more a day while the gingival symptom came out strongly. After the symptoms were partly reduced, a soft‐bristle toothbrush and inter‐dental brush were used. Chemotherapy with only bevacizumab was repeated an additional 10 times approximately every 3 weeks. According to the schedule for chemotherapy, the patient visited our dental office regularly. Every time, plaque and gingival evaluations were performed, and the subject received appropriate consultation about toothbrushing techniques and instruments, and finally, PMTC was carried out. The symptoms of gingiva were reduced gradually each time the number of visits increased, and regular toothbrushing and PMTC focusing on the subgingival area also became possible. Moreover, the fear of the bleeding of the gingiva moderated too. However, she had to wait several additional weeks from the end of chemotherapy for complete tolerance of inflammation including the local one in the oral cavity (Fig. 2).

**Figure 3:**
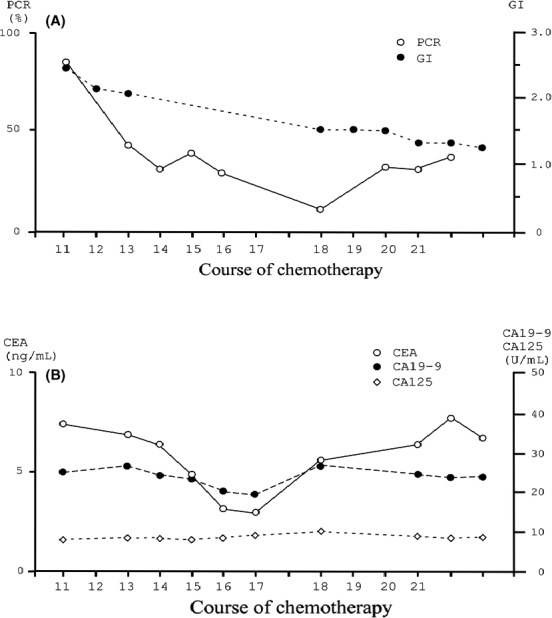
Dental values (A) and tumor markers (B) during chemotherapy with only bevacizumab, from the 11th course to after the therapy. PCR value (open circles) as index of dental plaque and GI value (closed circles) for assessment of gingival conditions are described in (A). Three tumor markers, which are CEA (open circle), CA19‐9 (closed circle), and CA125 (open rhombus), are measured, and the values are shown in (B). Numbers of horizontal bar described times of administration with bevacizumab. The periods between vertical small bars are approximately 3 weeks to each. The interval between 17th and 18th course are 6 weeks. The 21th course is the last administration of bevacizumab.

Gingival Index, PCR, and concentration of carcinoembryonic antigen (CEA), CA19‐9, CA125, numbers of white blood cells, and platelets in serum were monitored after the 11th course of chemotherapy (Fig. 3). GI and PCR decreased dramatically after the first visit to the dental office, and CEA decreased also simultaneously. Although CEA increased gradually after the 18th course, the value returned to the reference range 1 year later (CEA: ≤5.0 ng/mL), and recurrence of the cancer was not detected. Values of CA19‐9 and CA125 were within a reference range during whole chemotherapy (CA19–1: ≤37.0 U/mL; CA125: ≤35.0 U/mL). Numbers of white blood cells and platelets did not change significantly within the reference value (data not shown).

## Discussion

Oral adverse effects frequently emerge in chemotherapy for malignant lesions 14. In this report, a case subject who has been prescribed by bevacizumab for ovarian cancer complained of bleeding and swelling from gingival tissues, accompanied with pain. The symptoms have discouraged this patient from performing routine toothbrushing by herself. It is possible that accumulation of dental plaque, derived from impossibility of toothbrushing, as well as mucositis located at gingiva associated with bevacizumab, could induce severe inflammation in a synergistic manner.

In the first step of development of oral mucositis, epithelial cell thinning happens, followed by formation of ulceration. Oral bacteria could adhere and enter the lesion, and secondary infection could be established. Some risk factors relate to the frequency and severity of mucositis, such as the type of tumor involved, old age, poor oral hygiene, nutritional condition, and maintenance of kidney and liver function 14. It should be noted that mucositis can occur in the mucous membranes of the gastrointestine as well as the oral cavity. Chemotherapy‐induced mucositis usually occurs within 1 week after the first administration of chemotherapy and disappears in additional 2 weeks 15. No specific guideline has been published for effective treatment of mucositis induced by radiation as well as chemotherapy 16. In addition to local therapy, amifostine has been used for the prevention of oral mucositis from chemotherapy and radiation; however, there are conflicting results and insufficient data 17. The administration of amino‐acid‐rich elemental diet may be useful as a countermeasure for 5‐FU‐based chemotherapy‐induced oral mucositis in patients with colorectal cancer. In 19 of 22 patients, bevacizumab was prescribed in combination with the conventional chemotherapy. All cases, except for one, demonstrated assessment of excellent or good efficacy for prevention of oral mucositis 18.

Although there are not sufficient evidences for prevention of oral mucositis by oral hygiene protocols up to date, some reports described considerable benefits 17. The oral hygiene protocols contained regular oral assessments, toothbrushing, flossing, and frequent rinsing of the mouth as well as instruction of toothbrushing technique. In the article described above, it was explained that a decrease in microbiota in the oral cavity, derived from appropriate oral hygiene protocols, provides prevention of secondary infections, which result in reduction in exacerbation and incidence of mucositis. So, the main purpose of oral management should be put on decrease or reduction in mucositis, and prevention of incidence could be difficult. Meantime, other factors including nutrition, spicy food, smoking, and alcohol drinking were examined for effects on mucositis. Instead of rigorous approaches, there was no significant relationship to support that those factors play a role of incidence of mucositis 14.

The incidence of oral bleeding induced by bevacizumab has been reported rarely and has not been estimated sufficiently. Among 15 heavily pretreated patients with recurrent ovarian cancer, two patients had mild bleeding complications (epistaxis and gingival, grade I) 13. Although seven cases experienced bleeding of the gastrointestinal tract, and 33 cases did epistaxis in 67 patients with metastatic colorectal cancer, no cases experienced bleeding from gingiva. The epistaxis was transient and lasted less than five minutes. Four patients as a control group without prescription of bevacizumab (n = 35) also experienced the effect. There was no clear association with different kinds of therapy 19. A recent study revealed bleeding and hemorrhage emerged in 15 patients with ovarian cancer (n = 156); however, a detailed description about location was absent 20. Moreover, two of seven patients diagnosed with diffuse large B‐cell lymphoma complained of transient gum bleeding after prescription of bevacizumab combined with R‐CHOP regimen (rituximab, cyclophosphamide, doxorubicin, vincristine, and prednisone) 21.

In conclusion, the prescription of bevacizumab certainly induced gingival inflammation including swelling, bleeding, and pain during chemotherapy in a patient diagnosed with ovarian cancer and endometrial cancer. Moreover, the patient was afraid of trauma or worsening of symptoms by toothbrushing. This adverse effect has rarely been reported in comparison with other major effects, including hypertension, proteinuria, and thromboembolic events. The symptoms were reduced by professional oral hygiene protocols, which were instructed to the patient about toothbrushing using a soft brush and professional tooth cleaning, successfully and significantly. Although this adverse effect will disappeared after completion of chemotherapy, intervention of oral management would be important in the early stages of chemotherapy as much as possible, or before, to reduce gingival inflammation and to provide a feeling of security to patients during chemotherapy for more comfortable medical treatment.

## Conflict of Interest

All authors declare that they have no competing interest.

## Authorship

All authors contributed extensively to the work presented in this study. KM, MH, AD, and TS: performed clinical instruction and examination, interpreted the data, and wrote the manuscript. SS, TY, and TO: designed the study and wrote the manuscript.

## References

[1] Ferrero, J. M. , B. Weber , J. F. Geay , D. Lepille , H. Orfeuvre , M. Combe , et al. 2007 Second‐line chemotherapy with pegylated liposomal doxorubicin and carboplatin is highly effective in patients with advanced ovarian cancer in late relapse: a GINECO phase II trial. Ann. Oncol. 18:263–268. 17108151 10.1093/annonc/mdl376

[2] Cannistra, S. A. 2004 Cancer of the ovary. N. Engl. J. Med. 351:2519–2529. 15590954 10.1056/NEJMra041842

[3] Smolle, E. , V. Taucher , and J. Haybaeck . 2014 Malignant ascites in ovarian cancer and the role of targeted therapeutics. Anticancer Res. 34:l555–l561. 24692682

[4] Kim, K. J. , B. Li , K. Houck , J. Winer , and N. Ferrara . 1992 The vascular endothelial growth factor proteins: identification of biologically relevant regions by neutralizing monoclonal antibodies. Growth Factors 7:53–64. 1380254 10.3109/08977199209023937

[5] Burger, R. A. , M. F. Brady , M. A. Bookman , G. F. Fleming , B. J. Monk , H. Huang , et al. 2011 Incorporation of bevacizumab in the primary treatment of ovarian cancer. N. Engl. J. Med. 365:2473–2483. 22204724 10.1056/NEJMoa1104390

[6] Perren, T. J. , A. M. Swart , J. Pfisterer , J. A. Ledermann , E. Pujade‐Lauraine , G. Kristensen , et al. 2011 A phase 3 trial of bevacizumab in ovarian cancer. N. Engl. J. Med. 365:2484–2496. 22204725 10.1056/NEJMoa1103799

[7] Pujade‐Lauraine, E. , F. Hilpert , B. Weber , A. Reuss , A. Poveda , G. Kristensen , et al. 2014 Bevacizumab combined with chemotherapy for platinum‐resistant recurrent ovarian cancer: the AURELIA open‐label randomized phase III trial. J. Clin. Oncol. 32:1302–1308. 24637997 10.1200/JCO.2013.51.4489

[8] Fu, P. , Y. S. He , Q. Huang , T. Ding , Y. C. Cen , H. Y. Zhao , et al. 2016 Bevacizumub treatment for newly diagnosed glioblastoma: systematic review and meta‐analysis of clinical trials. Mol. Clin. Oncol. 4:833–838. 27123291 10.3892/mco.2016.816 PMC4840497

[9] Liu, X. , X. Liu , T. Qiao , W. Chen , and S. Yuan . 2016 Efficacy and safety of adding an agent to bevacizumab/taxane regimens for the first‐line treatment of Her2‐negative patients with locally recurrent or metastatic breast cancer: results from seven randomized controlled trials. Onco. Targets Ther. 9:3771–3781. 27445484 10.2147/OTT.S103954 PMC4938144

[10] Hurwitz, H. , L. Fehrenbacher , W. Novotny , T. Cartwright , J. Hainsworth , W. Heim , et al. 2004 Bevacizumab plus irinotecan, fluorouracil, and leucovorin for metastatic colorectal cancer. N. Engl. J. Med. 350:2335–2342. 15175435 10.1056/NEJMoa032691

[11] Sandler, A. , R. Gray , M. C. Perry , J. Brahmer , J. H. Schiller , A. Dowlati , et al. 2006 Paclitaxel‐carboplatin alone or with bevacizumab for non‐small‐cell lung cancer. N. Engl. J. Med. 355:2542–2550. 17167137 10.1056/NEJMoa061884

[12] Cannistra, S. A. , U. A. Matulonis , R. T. Penson , J. Hambleton , J. Dupont , H. Mackey , et al. 2007 Phase II study of Bevacizumab in patients with platinum‐resistant ovarian cancer or peritoneal serous cancer. J. Clin. Oncol. 25:5180–5186. 18024865 10.1200/JCO.2007.12.0782

[13] Chura, J. C. , K. Van Lseghem , L. S. Jr Downs , L. F. Carson , and P. L. Judson . 2007 Bevacizumab plus cyclophosphamide in heavily pretreated patients with recurrent ovarian cancer. Gynecol. Oncol. 107:326–330. 17706754 10.1016/j.ygyno.2007.07.017

[14] Chaveli‐López, B. , and J. V. Bagán‐Sebastián . 2016 Treatment of oral mucositis due to chemotherapy. J. Clin. Exp. Dent. 8:e201–e209. 27034762 10.4317/jced.52917 PMC4808317

[15] Epstein, J. B. , and M. B. Huhmann . 2012 Dietary and nutritional needs of patients after therapy for head and neck cancer. J. Am. Dent. Assoc. 143:588–592. 22653938 10.14219/jada.archive.2012.0237

[16] Sonis, S. T. 2004 A biological approach to mucositis. J. Support Oncol. 2:21–32. 15330370

[17] Lalla, R. V. , J. Bowen , A. Barasch , L. Elting , J. Epstein , D. M. Keefe , et al. 2014 MASCC/ISOO clinical practice guidelines for the management of mucositis secondary to cancer therapy. Cancer 120:1453–1461. 24615748 10.1002/cncr.28592 PMC4164022

[18] Ogata, Y. , N. Ishibashi , K. Yamaguchi , S. Uchida , H. Kamei , G. Nagayama , et al. 2016 Preventive effects of amino‐acid‐rich elemental diet Elental^®^ on chemotherapy‐induced oral mucositis in patients with colorectal cancer: a prospective pilot study. Support. Care Cancer 24:783–789. 26266658 10.1007/s00520-015-2844-0 PMC4689768

[19] Kabbinavar, F. , H. I. Hurwitz , L. Fehrenbacher , N. J. Meropol , W. F. Novotny , G. Lieberman , et al. 2003 Phase II, randomized trial comparing bevacizumab plus fluorouracil (FU)/leucovorin (LV) with FU/LV alone in patients with metastatic colorectal cancer. J. Clin. Oncol. 21:60–65. 12506171 10.1200/JCO.2003.10.066

[20] Selle, F. , G. Emile , P. Pautier , I. Asmane , D. G. Soares , A. Khalil , et al. 2016 Safety of bevacizumab in clinical practice for recurrent ovarian cancer: a retrospective cohort study. Oncol. Lett. 11:1859–1865. 26998090 10.3892/ol.2016.4146 PMC4774410

[21] Fu, Z. , J. Zhu , W. Zheng , W. Liu , Z. Ying , Y. Xie , et al. 2014 Safety and efficacy of bevacizumab combined with R‐CHOP regimen in seven Chinese patients with untreated diffuse large B‐cell lymphoma. Cancer Cell Int. 14:5. 24438119 10.1186/1475-2867-14-5 PMC3897913

